# The efficacy of micropulse laser combined with ranibizumab in diabetic macular edema treatment: study protocol for a randomized controlled trial

**DOI:** 10.1186/s13063-022-06593-2

**Published:** 2022-09-02

**Authors:** Xuejing Mi, Xiaoya Gu, Xiaobing Yu

**Affiliations:** Department of Ophthalmology, Beijing Hospital, National Center of Gerontology, Institute of Geriatric Medicine, Chinese Academy of Medical Sciences, Beijing, 100730 China

**Keywords:** Protocol, Diabetic macular edema, Anti-VEGF injections, Micropulse laser

## Abstract

**Background:**

At present, intraocular injection of anti-VEGF (vascular endothelial growth factor) drugs has replaced traditional laser therapy as the first-line treatment for DME (diabetic macular edema). However, ranibizumab, a commonly used anti-VEGF drug, is expensive and requires multiple intraocular injections. It places a heavy economic burden on patients with DME. Micropulse laser is safer than conventional laser and can reduce edema. Combined treatment with anti-VEGF may reduce the number of intraocular injections. This study will compare the efficacy of micropulse laser combined with ranibizumab treatment to ranibizumab monotherapy in the treatment of DME, providing a new regimen for future DME treatment.

**Methods:**

This study is a prospective randomized double-blind controlled clinical trial (RCT) in patients with DME. After 1-year follow-up, visual acuity and macular edema regression will be compared between micropulse laser combined with ranibizumab group and ranibizumab monotherapy group to determine whether the efficacy of micropulse laser combined with ranibizumab treatment was not lower than that of ranibizumab monotherapy in the treatment of DME. Visual acuity measured by the ETDRS chart is the primary outcome measure. The secondary outcome measures are CMT (central macular thickness) measured by OCT (optical coherence tomography) and the number of injections of two groups. Changes in visual acuity and CMT of the two groups will be compared at 12-month follow-up. Before patients are recruited, we provide them with informed consent, in which we explain to them the purpose and process of the study.

**Discussion:**

Micropulse laser combined with anti-VEGF drugs in the treatment of DME can reduce the number of intravitreal anti-VEGF injections, not only relieve the pain of the patients, but also ease the economic and psychological burden of patients, bringing great benefits. However, there is no treatment consensus for the parameters and specific methods of micropulse laser treatment for DME. There is a lack of clinical research data reference of micropulse laser combined with anti-VEGF therapy in clinical practice. This study intends to provide a new direction for clinical DME treatment and also provide a realistic consideration for the application of micropulse laser in DME treatment.

**Trial registration:**

ClinicalTrials.gov NCT03690947. Registered on 1 October 2018.

**Supplementary Information:**

The online version contains supplementary material available at 10.1186/s13063-022-06593-2.

## Administrative information

Note: the numbers in curly brackets in this protocol refer to SPIRIT checklist item numbers. The order of the items has been modified to group similar items (see http://www.equator-network.org/reporting-guidelines/spirit-2013-statement-defining-standard-protocol-items-for-clinical-trials/).

**Table:** 

Title {1}	The efficacy of micropulse laser combined with ranibizumab in diabetic macular edema treatment: study protocol for a randomized controlled trial
Trial registration {2a and 2b}.	ClinicalTrials.gov Identifier: NCT03690947
Protocol version {3}	Protocol version 1.0/2018.7.9.
Funding {4}	This study was funded by Capital clinical characteristic application research. Z181100001718079 and the project in Beijing Hospital (BJ-2019-154).
Author details {5a}	Xuejing Mi^1^, Xiaoya Gu^1^, Xiaobing Yu^1^. Corresponding author: Xiaobing Yu. ^1^Department of Ophthalmology, Beijing Hospital, National Center of Gerontology; Institute of Geriatric Medicine, Chinese Academy of Medical Sciences, P. R. China. Beijing 100730, China
Name and contact information for the trial sponsor {5b}	Investigator initiated clinical trial Xiaobing Yu, Email: yuxiaobing@sina.com
Role of sponsor {5c}	This is an investigator initiated clinical trial. Therefore, the funders played no role in the design of the study and collection, analysis, and interpretation of data and in writing the manuscript.

## Introduction

### Background and rationale {6a}

Diabetic retinopathy (DR) is one of the most common microvascular complications of diabetes, accounting for 13% of visual impairment in diabetic patients [1]. Diabetic macular edema (DME) is a common manifestation of DR and one of the major causes of visual loss in diabetic patients. There are many treatments for DME. Photocoagulation therapy, intravitreal anti-vascular endothelial growth factor (VEGF) injection, and intravitreal corticosteroid injection are its main methods [2]. The current first-line treatment for DME is an intravitreal injection of anti-VEGF agents [3], with ranibizumab being most commonly used in China. At present, the consensus of intravitreal ranibizumab injection is 3 consecutive monthly injections, followed by monthly follow-up and pro re nata (PRN) treatment. Several studies have shown that the number of injections within a year ranges from 7 to 8 [4]. Because ranibizumab is expensive and many DME patients suffer from recurrence, multiple intravitreal injections are needed, which brings a heavy economic and psychological burden to DME patients in China. Moreover, as the number of intravitreal injections increases, the risk of complications such as endophthalmitis also increases.

In previous studies of traditional macular grid photocoagulation combined with anti-VEGF therapy to treat DME, the timing of combined treatment was divided into immediate laser therapy and delayed laser therapy. Study results showed that combined treatment did not improve the visual acuity of patients or reduce the number of anti-VEGF injections and it left visible laser scars, causing damage to the retina, resulting in complications such as decreased visual acuity and reduced visual field. Therefore, the use of traditional photocoagulation is gradually decreasing. The 2017 European Union DME treatment guidelines stated that micropulse laser treatment is a promising treatment strategy for DME [5]. Micropulse laser is a type of subthreshold laser, which minimizes the damage of the neuroepithelial layer by reducing exposure time and energy. The guidelines stated that a micropulse diode laser emits a low-energy micropulse that confines the energy to the retinal pigment epithelium and avoids the outward spread of heat. Some randomized clinical studies have confirmed that the efficacy of micropulse diode laser treatment is comparable to that of traditional macular grid photocoagulation. However, it takes more time to achieve the same visual function and anatomical results [6, 7]. Therefore, guidelines recommended micropulse laser treatment for early diffuse DME patients with better visual acuity to avoid the spread of thermal energy and secondary chorioretinal injury. Compared to the previous diode micropulse laser, the new IQ577 nm micropulse laser has more obvious advantages. First, located in the inner and outer plexiform layers of the macula, lutein seldom absorbs yellow light. Therefore 577 nm yellow light is more suitable for the treatment of the macular than diode red light [8] with less damage. Second, treatment parameters such as duty cycle changed from 15 to 5% so that laser duration is shorter and the damage to the retina is less. Third, it is improved to a low-energy high-density grid laser with a better effect in eliminating edema. Previously, conventional lasers and other subthreshold lasers are forbidden within 500 μm diameter of the macula. But being able to treat in this range has become the most important innovation and advantage of the IQ577 nm micropulse laser. At present, some foreign studies and our own clinical experience have confirmed that 577 nm micropulse laser treatment for mild to moderate DME can effectively relieve edema and improve the visual function of patients [9]. The technique is simple and easy to learn, convenient for promotion, and more importantly, it can be easily accepted by patients for its cheap price.

To our knowledge, there were few prospective randomized controlled studies of micropulsed laser combined with anti-VEGF drugs in the treatment of DME. Only two retrospective studies published in 2017 and 2021 suggested that anti-VEGF drugs combined with the micropulse laser group could reduce the number of intravitreal injections compared to the anti-VEGF monotherapy group in the treatment of DME [10, 11].

### Objectives {7}

The purpose of this study is to evaluate the safety and efficacy of micropulse laser combined with intravitreal ranibizumab injections in the treatment of DME. Study subjects are 72 patients (more patients may be recruited) diagnosed with diabetic macular edema. By using a prospective randomized double-blind controlled study method, we hope to compare the changes of visual acuity and macular edema regression between micropulse laser combined with ranibizumab group with ranibizumab monotherapy group to determine whether the efficacy of micropulse laser combined treatment group in the treatment of DME is not lower than ranibizumab monotherapy and whether these two therapies are equally effective. Our goal is to reduce the number of intraocular injections and establish a new technique for the treatment of DME.

### Trial design {8}

**Fig. 1:**
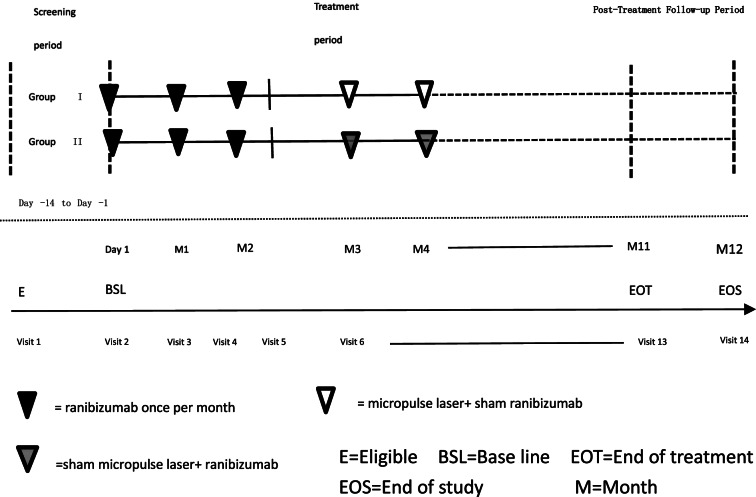
A flow chart of the study design

This study is a prospective, single-center, randomized controlled, double-blinded non-inferiority clinical trial. A flow chart of the study design is shown in Fig. 1. Study subjects are 72 patients (more patients may be recruited) diagnosed with diabetic macular edema.

#### Screening period: from day −14 to day −1

At screening (visit 1, between day −14 and day −1), after signing the informed consent (the model consent form is attached as a supplementary file), the patient is enrolled in the study and will undergo a series of examinations including ETDRS (Early Treatment Diabetic Retinopathy Study) visual acuity test, intraocular pressure measurement, slit-lamp, and indirect ophthalmoscopy, optical coherence tomography (OCT), optical coherence tomography angiography (OCTA), fundus fluorescein angiography (FFA), fundus color photography, vital signs, hematology, urinalysis, blood biochemistry, and glycosylated hemoglobin to assess eligibility for the study.

#### Treatment period: from day 1 to month 11

**Fig. 2:**
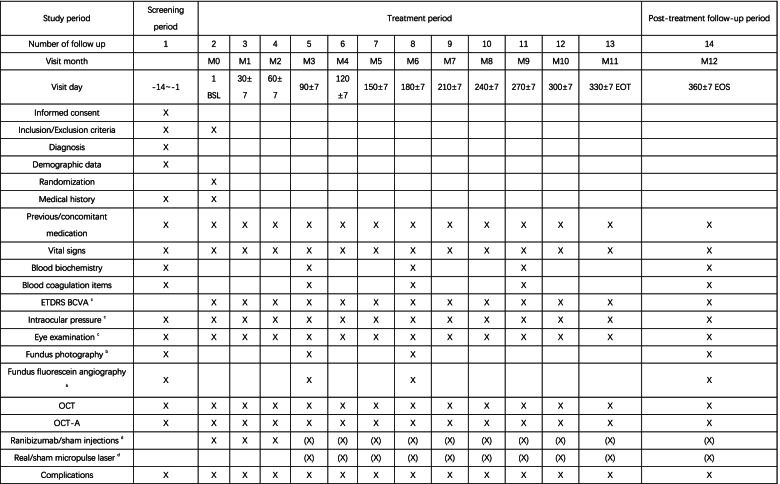
Assessment protocol from V1 to V14. *BSL *Base line, *EOT *End of treatment, *EOS *End of study. (X): Give treatment as needed. b: Except at the time of screening, at month 6 and at month 12 / end of the study, if the investigator needed information to evaluate the retreatment. These assessments are optional for therapeutic needs. c: Evaluation of both eyes. d: At 2 (±1) days after each study treatment, the investigator should contact the patient for a possible report of adverse events

At baseline (visit 2, day 1), eligible patients will be randomized by 1:1 ratio into micropulse laser combined with ranibizumab group or ranibizumab monotherapy group. After baseline visit, all patients receive 3 consecutive monthly ranibizumab injections. Subjects will then return to the study site on day 90 (± 7 days) and according to the retreatment criteria, patients will either receive micropulse laser and sham intravitreal ranibizumab or sham micropulse laser and intravitreal ranibizumab. Thereafter, subjects will return to the study site every 30 days (± 7 days) for assessment (schedule shown in Fig. 2) and accept appropriate treatment according to the retreatment criteria.

Group I (micropulse laser combined with ranibizumab group): 3 consecutive injections of intravitreal ranibizumab, followed by monthly observation, if:

A. Visual acuity decreases by greater than or equal to 5 ETDRS letters due to macular edema, then treat with micropulse laser therapy and sham intravitreal ranibizumab injection.
B. Visual acuity stays stable for at least two consecutive treatments (3 consecutive changes of no more than 5 letters), discontinue micropulse laser treatment.

Group II (ranibizumab monotherapy group): 3 consecutive injections of intravitreal ranibizumab, followed by monthly observation. According to the investigator’s judgment, if:

A. Visual acuity decreases by greater than or equal to 5 ETDRS letters due to macular edema, then treat with sham micropulse laser and intravitreal ranibizumab injection.
B. Visual acuity stays stable for at least two consecutive treatments (3 consecutive changes of no more than 5 letters), discontinue intravitreal ranibizumab injection.

#### Post-treatment follow-up period: from month 11 to month 12

For all patients, the last study assessment will occur at month 12 (1 month after the last possible micropulse laser or intravitreal ranibizumab injection in the study).

## Methods: participants, interventions, and outcomes

### Study setting {9}

In this study, we will enroll 72 patients (more patients may be recruited) diagnosed with diabetic macular edema in the Department of Ophthalmology, Beijing Hospital. Patients are men or women aged 30 to 80 years who are able to give informed consent.

### Eligibility criteria {10}

#### Inclusion criteria

Patients must meet all of the following criteria to be eligible for enrollment in the study:

1. Must sign an informed consent prior to performing any study-related assessments;
2. Male or female aged 30 to 80 years;
3. Diagnosed with type 2 diabetes, fasting blood glucose ≤ 10 mmol/L, glycosylated hemoglobin ≤ 10.0%;
4. Diabetic medications must be fixed within 3 months prior to Visit 1 and are expected to remain unchanged during the study;
5. Study eye must be diagnosed with non-proliferative diabetic retinopathy (NPDR), but not severe NPDR; the patient’s visual impairment was due to diabetic macular edema;
6. Best corrected visual acuity (BCVA) ≥ 24 and ≤ 78 ETDRS letters in the study eye at screening and baseline;
7. Presence of macular edema in the study eye shown on OCT examination with the central macular thickness (CMT) ≥300 um.
8. The study eye had not been treated with macular grid photocoagulation, intraocular surgery, intravitreal injection of corticosteroids or anti-VEGF drugs within 3 months prior to baseline;
9. BCVA ≥ 24 ETDRS letters of the fellow eye at screening;
10. The fellow eye has not been treated with anti-VEGF drugs within 3 months prior to baseline.

If both eyes are eligible, the eye with worse vision at screening will be selected as the study eye. Only the study eye will be treated with ranibizumab and/or micropulse laser.

#### Exclusion criteria

Patients who meet any of the following criteria are not eligible for inclusion in the study:

1. Inability to follow study or follow-up procedures;
2. Pregnant or lactating women and women with reproductive ability not taking effective contraceptive measures;
3. History of stroke or myocardial infarction within 3 months prior to screening;
4. Renal failure or creatinine level > 2.0 mg/dl;
5. Uncontrolled systemic diseases and treatments that may affect the study results;
6. Active ocular infection or intraocular inflammation in any eye;
7. Iris neovascularization or neovascular glaucoma in any eye;
8. History of uveitis or vitreomacular traction in any eye;
9. Glaucoma or intraocular pressure (IOP) ≥ 24 mmHg in the study eye.

### Who will take informed consent? {26a}

Potential trial participants will be provided with an informed consent drafted by the primary investigator in a reception room. A sub-investigator will explain the purpose and process of the study in detail. After fully understanding their rights and obligations, risk and benefits, potential trial participants, or authorized surrogates will sign two identical informed consent forms. One will be kept by the study group, the other one will be kept by the participant.

### Additional consent provisions for collection and use of participant data and biological specimens {26b}

Not applicable, there are no additional consent provisions, and this trial does not involve collecting biological specimens for storage for use in the current study.

## Interventions

### Explanation for the choice of comparators {6b}

We used BCVA and CMT as the main outcome measures. BCVA is a functional indicator and effective treatment is to improve or maintain patients’ vision. CMT is often reduced before visual acuity improvement and could be used as a morphological indicator.

### Intervention description {11a}

Micropulse laser combined with ranibizumab group (group I): After three consecutive intravitreal injections of 0.5mg ranibizumab, patients are followed-up monthly in Beijing Hospital. If visual acuity decreases by greater than or equal to 5 ETDRS letters due to macular edema, then treat with micropulse laser (the treatment parameters we apply are 577nm micro-pulse laser, 200μm spot size, 5% duty cycle of 0.2 seconds, 400mW power, 7×7 grid, panmacular treatment) and sham intravitreal ranibizumab injection. And if visual acuity stays stable for at least two consecutive treatments (3 consecutive changes of no more than 5 letters), then discontinue micropulse laser treatment.

Ranibizumab monotherapy group (group II): After three consecutive intravitreal injections of 0.5mg ranibizumab, patients are followed-up monthly in Beijing Hospital. Sham micropulse laser and intravitreal ranibizumab injection will be performed as needed if visual acuity decreases by greater than or equal to 5 letters due to macular edema. If visual acuity stays stable for at least two consecutive treatments (3 consecutive changes of no more than 5 letters), discontinue intravitreal ranibizumab injection, then discontinue intravitreal ranibizumab injection.

### Criteria for discontinuing or modifying allocated interventions {11b}

Treatment of the study eyes will be based on the trial design and retreatment criteria. In case of severe vision loss (visual acuity decreased ≥15 letters compared with the last follow-up) due to exacerbation of DME, or severe adverse reactions, or participants’ request, subjects will be arranged to discontinue and leave the study and then continue with the next symptomatic treatment.

### Strategies to improve adherence to interventions {11c}

During the follow-up period, investigators will be arranged to respond to patient inquiries. Investigators will contact patients 2 (± 1) days before each visit to improve adherence to intervention protocols.

### Relevant concomitant care permitted or prohibited during the trial {11d}

Not Applicable. No relevant concomitant care is prohibited during the trial.

### Provisions for post-trial care {30}

Follow-up visits at the outpatient clinic will be arranged and patients will receive post-trial treatment plans. If patients suffer harm from trial participation. compensation will be made by Beijing Hospital in accordance with relevant laws and regulations.

### Outcomes {12}

Assessment protocol for the current study from V1 to V14 is provided in Fig. 2.

#### Primary outcome measure

BCVA: Best corrected visual acuity is measured using standard ETDRS protocol at baseline and every follow-up visit. Changes in visual acuity will be compared between the two groups at a 12-month follow-up.

#### Secondary outcome measures

CMT: OCT is used to measure CMT at baseline and every follow-up visit. Changes of CMT will be compared between the two groups at 12-month follow-up.

Number of injections: Number of intravitreal injections of two groups will be compared at a 12-month follow-up.

### Participant timeline {13}

A flow chart of the study design is shown in Fig. 1.

1. Screening period: from day −14 to day −1
2. Treatment period: from day 1 to month 11
3. Post-treatment follow-up period: from month 11 to month 12

### Sample size {14}

Using two-sided test, with αas 0.05, changes of visual acuity as less than 5 letters between two groups, standard deviation as 6.53 (REVEAL study [12] as reference), enrolling 56 cases of subjects can set the power to 90%. Considering drop out, set the lost follow-up rate as 20%, then the final sample size is calculated as 72 subjects, with 36 subjects in micropulse laser combined with ranibizumab group and 36 subjects in the ranibizumab monotherapy group.

### Recruitment {15}

When physicians meet qualified patients in the outpatient department of ophthalmology, they will refer them to the investigator’s team who are specialized in screening. Sub-investigatiors will then introduce the study to the patients.

## Assignment of interventions: allocation

### Sequence generation {16a}

A table of random numbers will be used for randomizing, Patient number 1 to 72 will each be assigned a random number by using a random number table. Then they are put in numerical order from small to large, the first 36 will be group I (micropulse laser combined with ranibizumab group), and the other 36 will be group II (ranibizumab monotherapy group).

### Concealment mechanism {16b}

The randomization results of patients are put in sealed envelopes. At visit 5 (3-month follow-up), unblinded investigators will open the envelopes to go through interventions (intravitreal injections or micropulse laser or sham treatment).

### Implementation {16c}

The unblinded investigators will generate the allocation sequence and assign participants to interventions. The blinded investigators will enroll participants.

## Assignment of interventions: Blinding

### Who will be blinded {17a}

Investigators responsible for follow-up evaluation, technicians who operate OCT, and data analysts are blinded. When intervention is required, the patient will either receive intravitreal injections plus sham micropulse laser treatment, or micropulse laser plus sham intravitreal injections. Therefore trial participants are blinded throughout the study. Only the investigators qualified for laser treatment and intravitreal injections are unblinded.

### Procedure for unblinding if needed {17b}

In case of severe vision loss due to exacerbation of DME and requiring additional treatment or severe adverse reactions, subjects will be arranged to leave the study. The subjects who leave the group will be unblinded.

## Data collection and management

### Plans for assessment and collection of outcomes {18a}

Professional technicians will be responsible for FFA, OCT examination, and visual acuity examination. OCT was performed using Spectralis (Spectralis HRA+OCT; Heidelberg Engineering, Heidelberg, Germany). FFA was performed using HRA2 (Heidelberg Retinal Angiograph, Heidelberg Engineering, Heidelberg, Germany).

### Plans to promote participant retention and complete follow-up {18b}

Investigators will contact patients 2 (± 1) days before each visit to promote patients’ retention and complete follow-ups. And we will also arrange follow-up visits at the outpatient clinic to support patients with subsequent treatment if they decide to drop out of the study.

### Data management {19}

Data such as BCVA, IOP, and adverse events will be uploaded to the Electronic Data Capture (EDC) system.

### Confidentiality {27}

Patients will be numbered instead of their names. Personal information will be collected through these numbers. We will keep patient study records confidential as required by law. Relevant laws in our country provide guarantees for the security of privacy, data, and authorized access. The name, ID number, address, telephone number, or any information that can directly identify the patient in the study records will not be disclosed outside the Beijing Hospital unless required by relevant laws.

### Plans for collection, laboratory evaluation, and storage of biological specimens for genetic or molecular analysis in this trial/future use {33}

Not applicable. There are no biological specimens.

## Statistical methods

### Statistical methods for primary and secondary outcomes {20a}

The randomized set will consist of all randomized patients.

Full Analysis Set (FAS): Consists of all patients in the randomized set who are assigned to study treatment. They will be analyzed according to the treatment assigned to the subjects at randomization and the intention-to-treat principle.

Per Protocol Set (PPS): Consists of all patients in the full analysis set who complete the trial, had no significant protocol violations, and had no events that would confound the interpretation of the analysis. The analysis of the primary variable will be performed on the full analysis set.

For continuous and ordered categorical variables, analysis of variance (ANOVA) or analysis of covariance models which are both with baseline assessment as covariates were used to compare the changes between each treatment group and the baseline. Stratified and unstratified Cochran Mantel-Haenszel tests were also considered.

### Interim analyses {21b}

The research committee of capital clinical characteristic application research and Beijing Hospital will meet over one year after the study started enrolling as the interim evalution. If assessment objectives are met, the study may continue.

### Methods for additional analyses (e.g., subgroup analyses) {20b}

Stratification will be made according to DME type and baseline BCVA classification: focal vs. diffuse, baseline BCVA ≤ 60 letters vs. > 60 letters.

### Methods in analysis to handle protocol non-adherence and any statistical methods to handle missing data {20c}

For missing values, the analysis will follow the LOCF (last observation carried forward) approach to obtain approximate results. If the patient dropped out, data of the last post-baseline observation during follow-up will take place of the missing data. If there were intermediate missing values (i.e., one missed visit during follow-up, then the patient resumed the visit), these values will be replaced with the mean value of the previous observation and the first subsequent observation.

### Plans to give access to the full protocol, participant-level data and statistical code {31c}

Public access to the full protocol and dataset can be acquired by logging on to ClinicalTrials.gov.

## Oversight and monitoring

### Composition of the coordinating center and trial steering committee {5d}

This study is funded by Capital clinical characteristic application research and Beijing Hospital. During the study period, the research committee in both institutions will meet over at the beginning, the interim and the end of the trial to check the completeness of patient records,compliance with the study protocol and GCP, enrollment progress, and to ensure that the storage, distribution, and counting of the study drugs are in compliance with requirements.

### Composition of the data monitoring committee, its role, and reporting structure {21a}

We have the original medical records and the Beijing Municipal Science and Technology Commission will conduct regular assessment. No data monitoring committee is needed.

### Adverse event reporting and harms {22}

Investigators will contact patients 2 (±1) days after each study treatment to obtain a report of possible adverse events. All adverse events are recorded in the EDC system. If severe adverse events occur, the Beijing Hospital ethics committee will be informed within 48 hours.

### Frequency and plans for auditing trial conduct {23}

Auditing process will be held at the interim and end of the study, independent from investigators and the sponsor.

### Plans for communicating important protocol amendments to relevant parties (e.g. trial participants, ethical committees) {25}

If any protocol amendments are made, the informed consent form needs to be overwritten and submitted for approval by the ethics committee. Trial participants will need to resign the informed consent form. Any deviations and violations from the Protocol will be fully documented, such as failing to withdraw subjects from the study in accordance with the discontinuation criteria; giving incorrect treatment doses; giving drug combinations prohibited by the protocol, etc.

## Dissemination plans {31a}

Patients will be informed of study results in the outpatient department or by phone after the study’s article is published.

## Discussion

Ranibizumab is a recombinant humanized monoclonal antibody with specificity for VEGF [13]. It is essentially an antibody fragment of bevacizumab [14]. The conventional treatment for DME is the intravitreal injection of ranibizumab with the 3+PRN regimen, that is, three consecutive monthly intravitreal injections for 3 months, followed by monthly re-examinations and as-needed treatment.

Micropulse laser acts on retinal pigment epithelium (RPE) cells without producing macroscopic laser spots or scars. Compared to the previous diode micropulse laser, the new IQ577 nm micropulse laser has more obvious advantages. First, located in the inner and outer plexiform layers of the macula, lutein seldom absorbs yellow light. Therefore 577 nm yellow light is more suitable for the treatment of the macula than diode red light [8] with less damage. Second, treatment parameters such as duty cycle changed from 15 to 5% so that laser duration is shorter and the damage to the retina is less. Third, it is improved to a low-energy high-density grid laser with a better effect in eliminating edema. Previously, conventional lasers and other subthreshold lasers are forbidden within 500 μm diameter of the macula. But being able to treat in this range has become the most important innovation and advantage of the IQ577 nm micropulse laser. At present, some foreign studies and our own clinical experience have confirmed that 577 nm micropulse laser treatment for mild to moderate DME can effectively relieve edema and improve the visual function of patients [9]. The technique is simple and easy to learn, convenient for promotion, and more importantly, it can be easily accepted by patients for its cheap price. Over recent years, it has been suggested that micropulse laser combined with intravitreal anti-VEGF drugs in the treatment of DME may reduce the number of intravitreal injections [11]. It can also significantly reduce CMT and improve BCVA for refractory and mild DME [15, 16]. However, there is no treatment consensus for the parameters and specific methods of micropulse laser treatment for DME. And there are no guidelines for the timing and treatment parameters of micropulse laser combined with anti-VEGF therapy that can be used for reference. There was only two retrospective studies published in 2017 and 2021 that suggested that if anti-VEGF drugs are used to treat DME, combine micropulse laser treatment after macular edema reduces to mild to moderate, then the number of intravitreal injections can be reduced compared to anti-VEGF monotherapy group [10, 11]. The results of these studies provided the groundwork for our study.

Micropulse laser combined with anti-VEGF drugs in the treatment of DME can reduce the number of intravitreal anti-VEGF injections, not only relieve the pain of patients, but also ease the economic and psychological burden of patients, bringing great benefits. However, there is no treatment consensus for the parameters and specific methods of micropulse laser treatment for DME. There is a lack of clinical research data reference of micropulse laser combined with anti-VEGF therapy in clinical practice. This study intends to provide a new direction for clinical DME treatment and also provide a realistic consideration for the application of micropulse laser in DME treatment.

## Trial status

Protocol version 1.0/2018.7.9. Recruitment began in January 2019 and completed in August 2021.

We finished this manuscript before August 2021, but it was rejected after being revised by other journals. After that, I had to prepare for the graduation exam, so we did not get time to work on it and submit it early. Our recruitment should have been completed in August last year, but due to the COVID-19 prevention and control policies, some patients were lost to follow-up, so we had to recruit new patients in the past several months. Recruitment will be completed if all the patients we recruited can be followed up. But if they cannot be followed up again due to COVID-19, we will have to recruit new patients who live in Beijing. The last visit should be at the end of this year if all goes well.

## Supplementary Information

**Additional file 1.** Informed consent form.

## Data Availability

Primary investigator and sub-investigators who signed contractual agreements will have access to the final trial dataset.

## Abbreviations

DME: Diabetic macular edema
VEGF: Vascular endothelial growth factor
RCT: Randomized double-blind controlled clinical trial
CMT: Central macular thickness
OCT: Optical coherence tomography
DR: Diabetic retinopathy
PRN: Pro re nata
ETDRS: Early Treatment Diabetic Retinopathy Study
OCTA: Optical coherence tomography angiography
FFA: Fundus fluorescein angiography
NPDR: Non-proliferative diabetic retinopathy
BCVA: Best corrected visual acuity
CRT: Central retinal thickness
IOP: Intraocular pressure
EDC: Electronic Data Capture
FAS: Full Analysis Set
PPS: Per Protocol Set
LOCF: Last observation carried forward
ANOVA: Analysis of variance
RPE: Retinal pigment epithelium

## References

[1] Klein R, Lee KE, Gangnon RE, Klein BE (2010). The 25-year incidence of visual impairment in type 1 diabetes mellitus the Wisconsin epidemiologic study of diabetic retinopathy. Ophthalmology..

[2] Group of fundus Diseases, Ophthalmology Society, Chinese Medical Association (2014). Clinical guidelines for diabetic retinopathy in China. Chin J Ophthalmol.

[3] Lang GE, Berta A, Eldem BM, Simader C, Sharp D, Holz FG (2013). Two-year safety and efficacy of ranibizumab 0.5 mg in diabetic macular edema: interim analysis of the RESTORE extension study. Ophthalmology..

[4] Elman MJ, Aiello LP, Beck RW, Bressler NM, Bressler SB, Diabetic Retinopathy Clinical Research N (2010). Randomized trial evaluating ranibizumab plus prompt or deferred laser or triamcinolone plus prompt laser for diabetic macular edema. Ophthalmology..

[5] Schmidt-Erfurth U, Garcia-Arumi J, Bandello F, Berg K, Chakravarthy U, Gerendas BS (2017). Guidelines for the Management of Diabetic Macular Edema by the European Society of Retina Specialists (EURETINA). Ophthalmologica..

[6] Lavinsky D, Cardillo JA, Melo LA, Dare A, Farah ME, Belfort R (2011). Randomized clinical trial evaluating mETDRS versus normal or high-density micropulse photocoagulation for diabetic macular edema. Invest Ophthalmol Vis Sci.

[7] Figueira J, Khan J, Nunes S, Sivaprasad S, Rosa A, de Abreu JF (2009). Prospective randomised controlled trial comparing sub-threshold micropulse diode laser photocoagulation and conventional green laser for clinically significant diabetic macular oedema. Br J Ophthalmol.

[8] Mainster MA (1986). Wavelength selection in macular photocoagulation. Tissue optics, thermal effects, and laser systems. Ophthalmology..

[9] Mansouri A, Sampat KM, Malik KJ, Steiner JN, Glaser BM, Medscape. (2014). Efficacy of subthreshold micropulse laser in the treatment of diabetic macular edema is influenced by pre-treatment central foveal thickness. Eye (Lond).

[10] Altinel MG, Acikalin B, Alis MG, Demir G, Mutibayraktaroglu KM, Totuk OMG (2021). Comparison of the efficacy and safety of anti-VEGF monotherapy versus anti-VEGF therapy combined with subthreshold micropulse laser therapy for diabetic macular edema. Lasers Med Sci.

[11] Moisseiev E, Abbassi S, Thinda S, Yoon J, Yiu G, Morse LS (2018). Subthreshold micropulse laser reduces anti-VEGF injection burden in patients with diabetic macular edema. Eur J Ophthalmol.

[12] Ishibashi T, Li X, Koh A, Lai TY, Lee FL, Lee WK (2015). The REVEAL Study: ranibizumab monotherapy or combined with laser versus laser monotherapy in Asian patients with diabetic macular edema. Ophthalmology..

[13] van Wijngaarden P, Coster DJ, Williams KA (2005). Inhibitors of ocular neovascularization: promises and potential problems. JAMA..

[14] Steinbrook R (2006). The price of sight--ranibizumab, bevacizumab, and the treatment of macular degeneration. N Engl J Med.

[15] Akhlaghi M, Dehghani A, Pourmohammadi R, Asadpour L, Pourazizi M (2019). Effects of subthreshold diode micropulse laser photocoagulation on treating patients with refractory diabetic macular edema. J Curr Ophthalmol.

[16] Citirik M (2019). The impact of central foveal thickness on the efficacy of subthreshold micropulse yellow laser photocoagulation in diabetic macular edema. Lasers Med Sci.

