# Supplementary material for: The efficacy of micropulse laser combined with ranibizumab in diabetic macular edema treatment: study protocol for a randomized controlled trial
Source: Trials. 2022 Sep 2;23:736. doi: 10.1186/s13063-022-06593-2 (PMC9438173; doi:10.1186/s13063-022-06593-2)
Supplement: Supplementary file 1 — Additional file 1. Informed consent form. [file 13063_2022_6593_MOESM1_ESM.pdf]

# Informed consent form

## A randomized controlled trial of micropulse laser combined with ranibizumab in the treatment of diabetic macular edema

Because you are eligible to participate in a randomized, controlled trial of micropulse laser plus ranibizumab for diabetic macular edema, you are invited to participate in this study.Please read this informed consent form carefully and make your decision to participate in the study with caution.The research doctors or researchers are more than willing to discuss and explain what you don't understand about the informed consent.Before you make a decision about whether to participate in this study, you can fully discuss it with your family and friends.If you are participating in another study, be sure to tell your study physicians or investigators.

This study, conducted by Beijing Hospital Yu Xiaobing, was funded by the Beijing Municipal Science and Technology Commission.

**1.Why was this study conducted?**

**1.1 Background**: Diabetes Mellitus (DM) is a common disease in developed countries, with an estimated prevalence of 8.3% worldwide.Diabetic Retinopathy (DR) is one of the most common microvascular complications, accounting for 13% of visual impairment in Diabetic patients. Diabetic Macular Edema (DME) is a common manifestation of DR. It is a major cause of vision loss in DM patients.Currently, there are many studies on treatment options for DME. Early standard treatment was macular focal/grille laser photocoagulation, but conventional laser therapy did not improve visual acuity for the most part and remaining visible laser spots on the retina, causing damage to the retina, resulting in decreased vision, reduced field of vision and choroidal neovascularization. Recently, intraocular injection of anti-VEGF drugs has been widely used in the treatment of DME, and either injection alone or combined with focal/grating laser photocoagulation is more effective than laser photocoagulation alone.The efficacy of ranibizumab in the treatment of DME is positive. However, due to the high price of ranibizumab, monthly continuous intraocular injection of ranibizumab brings heavy economic and mental burden to Chinese patients.Therefore, there is an urgent need for a new type of laser with

less tissue damage to treat DME.As a novel laser, 577nm micropulse laser selectively targets the retinal pigment epithelium (RPE) to segment the continuous wave into a series of repeated short pulses to avoid tissue damage caused by short-term heat accumulation.There was no visible laser spot in the eye, and the damage was extremely minimal.At the same time, the excitation induces the production of many cytokines, which in turn attenuates macular edema.

**1.2 Study objectives**: The aim of this study is to investigate the efficacy, number of treatments, and safety of micropulse laser photocoagulation combined with intraocular injection of ranibizumab in the treatment of diabetic macular edema.The results of this study will provide guidance for the treatment of diabetic macular edema in the future.

**1.3 Study type**: This study was a prospective single-center randomized double-blind controlled study.

**2. How many people will participate in the study?**

About 70 participants will participate in the study at Beijing Hospital

**3. How long will the study last?**

The study period was 3 years, and you were followed until 12 months after the index treatment.

**4. Is participation in the study voluntary?**

Your participation in the study is voluntary and there will be no penalties or loss of benefits if you refuse to participate.

**5. How was the study conducted?**

If you provide this informed consent to participate in the study, you will enter the study process as follows:

**5.1 screening**

Physical examination and medical history;

Vital signs (e.g., breathing, body temperature, pulse, etc.)

Blood test, intraocular fluid cytokine test (optional);

Specialized ophthalmology examination (visual acuity, intraocular pressure, slit-lamp, optical coherence tomography, optical coherence tomography angiography, fundus fluorescent angiography, fundus color)

**5.2 Grouping/randomization**

In this study, you will be randomly assigned into the following one of the two treatment groups: respectively is licensed separately in treatment group and licensed joint micropulse laser photocoagulation treatment group.You have a 50% chance of entering the combination-therapy group.The study lasted approximately 12 months.

**5.3 Study measures/Procedures Therapeutic Drugs:**

Ranibizumab treatment involves the injection of 0.5mg of ranibizumab into the vitreous of your eye.This will take about 10 minutes and you will be lying in bed.Because before the injection, will give the local anesthetic, as a result, although you may feel a slight pressure, but not expected to feel the pain. Ranibizumab treatment would be administered by ophthalmologic students with qualifications and experience in intraocular injection.

Laser photocoagulation:

They will sit in front of a slit lamp in the laser treatment room and receive laser treatment.A local anesthetic is dripped into your eye to anesthetize the ocular surface, then a special contact lens is placed on the eye to keep the eyelid open and focus the laser beam on your retina.After treatment, the contact lens is removed.Laser therapy focuses beams of light on your retina as small spots of light.During treatment, you may see flashes of light.It usually doesn't cause pain.Treatment will take about 5 to 10 minutes.Your vision may become blurred after laser treatment.Vision should become clear after 3 to 4 hours.In order to keep the blind state, will use false to simulate laser photocoagulation therapy, namely the eye will not accept the active laser beam exposure.

False treatment is necessary, to ensure that the results of this study is not affected by understanding of treatment.

According to routine clinical diagnosis and treatment, the doctor can guide you in every time before each visit and glass body within a few days after injection, at home to use antibiotics eye drops (the eyes).

In this study are not allowed to use the following for eye treatment: Other anti-VEGF agents in addition to the study drug and  intraocular corticosteroids.

The research program

If you take part in this study, throughout the study period, will need you to research center visit 14 times.According to the application of the research, visit may take four to six hours at a time.Research will continue for about one year, including 14 days (the) stage screening, 11 months of treatment and follow-up of 1 month.When your study

doctor think you describe the symptoms of need further checks, you may need to outside of the regular plan to visit research center.

First visit: if you agree to participate in this study, and have already signed the informed consent, in the time of the study, will be for you "screening tests", in order to determine whether you fit to participate in this study."Screening tests”include:

Obtain your demographic information health status and medical history

Determine medications you have used or are using measure your pulse and blood pressure as well as your height and weight

Binocular eye tests, including visual acuity (to test your visual acuity) intraocular pressure slit-lamp microscopy and dilated retinal examination Optical coherence tomography (OCT, non-invasive test, checks macular thickness) Optical coherence tomography angiography (OCTA, checks macular blood flow) Color fundus photography (taking pictures of the retina) Fluorescence angiography (injecting a dye into a vein in your arm and taking a picture of a specific fundus)

Blood and urine samples will be collected for laboratory analysis.

Treatment visit (visit 2 to 13) : "screening inspection is completed, if the doctor make sure you fit to participate in this study, please on the 14 days for the first time treatment supervision(Day 1, Visit 2), and you will be randomly assigned to one of the two treatment groups to receive the appropriate treatment.

You will be required to visit the study center once a month for the next 11 months. The contents of each visit include:

Ask about your health status and any changes in your medication.

Measure pulse and blood pressure.

Ophthalmic examination of the study eye: including intraocular pressure optical coherence tomography (IOP), optical coherence tomography (OCT), angiography, slit-lamp microscopy (SLIT-lamp microscopy) and dilated retinal examination before and after the study.Your study physician will also perform fluorescein angiography and color fundus photography if necessary. (mandatory at visits 1 and 14).

And check your vision acuity after you receive the study treatment.

Optical coherence tomography and vision acuity examination of the non-study eyes (contralateral eyes) will also be performed at visit 7 (month 6).

At each visit, a routine ophthalmic examination was performed on both eyes.

Depending on which treatment group you are in, you will receive ranibizumab injection at visits 2, 3, and 4, and micropulsed laser photocoagulation or sham

m icropulsed laser photocoagulation treatment at visit 5.

Study end visit (visit 14 days or within 30 days of last study treatment)

If you have completed study treatment or if you withdraw early from the study, you will receive an end-of-study visit. The end of study visit includes the following contents:

Ask about your health and your medication situation whether there is any change .

Measure your pulse and blood pressure, height and weight.

The eye exam: including visual acuity intraocular pressure optical coherence tomography optical coherence tomography and mydriatic retinal vascular imaging slit lamp microscope examination.Fluorescence fundus angiography and photography.

Blood and urine samples will be collected for laboratory analysis.

No study treatment will be given during this visit.

**6. What are the risks of participating in this study?**

Participation in this study may confer risks to you as follows.You can discuss these risks with your study physician.

Intraocular injection may cause the following adverse reactions, You may experience all, some, or none of the following adverse reactions.

Relatively common (incidence more than 10%):

Eye congestion and bleeding;eye pain;sensation of small particles or spots (floating objects) in the eyes;increased intraocular pressure;ocular irritation;foreign body sensation of the eye;visual impairment;inflammation or infection of the eyelid rim (blepharitis);tearing;redness of the eye; dry eye.

Relatively infrequent:

Blindness;Intraocular infection and inflammation;anterior atrial abscess (accumulation of white blood cells in the front of the eye);anterior atrial hemorrhage (accumulation of blood in the front of the eye);injection site pain;injection site irritation intraocular paresthesia and eyelid irritation;stroke.

The potential risk of laser treatment.

Macular edema is not fade.

The risk of blood.

The risk of blood from the arm include brief discomfort and/or blue.Although the possibility is very small, also possible infection, bleeding, clotting or syncope.

Drug interactions.

Before the start of the study on the safe side, you must tell the doctor or nurse all you are taking prescription drugs, traditional Chinese medicine products, over-the-counter medications, vitamins and natural supplements and other health care products.Before taking these drugs are needed in the process of research also please be sure to tell your study doctor or nurse.

Other risks: There may also be risks, discomfort, drug interactions or adverse reactions that are not currently foreseen.

**7. What are the benefits of participating in the study?**

If you agree to participate in the study, you will receive free ranibizumab and free macular and peripheral retinal laser photocoagulation to improve or stabilize your vision during the study.Your participation may help other patients receive better care in the future.

**8. Are there any medical options available to me other than participating in the study (or if NOT)?**

In addition to participating in the study, you have the following options:

Intravitreal injection of corticosteroids

Intravitreal injection of other anti-VEGF drugs

laser treatment

You can ask your doctor about the potential benefits and risks of these treatments.

**9. Will my information be kept confidential?**

We will keep your research records confidential as required by law. China's relevant laws provide security for privacy data and authorized access. Unless required by relevant laws, your name, ID number, address, telephone number or any information in the research records that can directly identify you will not be disclosed to Beijing Hospital.

**Who has access to the research records, and to whom is this information publicly available?**

As part of the study, Yu XiaoBing doctors and her team will let you perform certain tests.Some of the blood and urine samples and imaging examination is your part of the routine medical treatment.She will use these results to treat you And completed the study.These results will record in your medical records, and report to a Beijing hospital.As for unconventional, inspection and the results of this study will be written into your medical records.To ensure that the study complies with relevant legal and regulatory requirements, your records may be reviewed.Reviewers included the ethics review board.When reviewing your study records, they may need to review all of your medical records.

Your identity will not be disclosed when the information and data obtained from this study are published in scientific meetings or scientific journals

**10. Do I have to pay for the study?**

Costs and Reimbursement for Participation in the study: You are not responsible for the cost of the study drug or for any ophthalmic examination or laser treatment performed solely for research purposes.

**11. What compensation can I get?**

At the last visit before the end of the trial, if you had a substantial worsening of macular edema resulting in a substantial loss of vision, you were compensated, at the discretion of the investigator, with a free dose of ranibizumab.

**12. What if research-related injuries occur?**

It is important that you carefully follow all guidance provided by the study physicians and study staff.

If a study-related injury occurred during the study, the Beijing Hospital provided immediate care.Beijing Hospital will compensate for research-related injuries in accordance with relevant laws and regulations in China.

If you have questions related to the study or study-related injuries, please contact Xiaobing Yu at 010-85132171 during weekdays and at 15699847843 during after-hours, weekends, or holidays.

**13. Do I have the right to withdraw from the study?**

Your participation in the study is voluntary, and you may withdraw your consent and withdraw from the study at any time without any reason.

If you decide to withdraw from the study midway through the study, contact Dr. Yu in writing to let her know that you will withdraw from the study.Her mailing address is Department of Ophthalmology, Beijing Hospital, 1 Dahua Road, Dongdan, Beijing.

If Dr. Yu feels that stopping the medication suddenly would affect your health, she may ask you to come to the hospital for an examination before stopping the medication.

For your own safety, she may also ask you to do some of the tests you would normally do when completing your study.

In principle, after your withdrawal, the investigator will maintain your information until it is destroyed and will not continue to use or disclose this information.However, in the following rare circumstances, investigators will continue to use or disclose information about you, even if you have withdrawn from the study or the study has ended.These include:

Removal of your information will affect the results of the study;

Provide limited information for research, teaching or other activities (this information does not include your name, identification number, or other personal information that identifies you);

When ethics committees and government regulators need to monitor a study, they will request access to all study information, including information about your current participation in the study.

**14. Will information be available in a timely manner that may affect continued participation in the study**

During the study, we will provide you or your legal representative with some new information that may affect your willingness to continue to participate in the study for your health benefits

**15. Is it possible to terminate the study?**

If your condition worsens, or if you have a serious adverse reaction, or if your research physician decides that continuing to participate in the study is not in your best interest, she may decide to withdraw you.Reasons for this were the use of anti-VEGF agents during treatment, systemic medications known to be toxic to the lens, retina, or optic nerve, including deferoxamine, chloroquine/hydroxychloroquine, tamoxifen, ethambutol, and phenol-thiazides, any type of trial drug, or trial treatment.We will inform you promptly if this occurs, and your research physician will discuss your options with you.

**16. Is there a sample storage option?**

Do you consent to the storage of your blood and intraocular fluid for optional cytokine detection studies in this study?
yes no
If you agree to store your blood and intraocular fluid, please select:
Cytokines can be used after testing research, but need to ask for my consent.
Cytokines can be used after testing study, don't have to ask for my opinion, authorize the ethics committee.

Can't be used in any other research.

Other choice.

You can change this decision at any time in the future.At that time, please contact Dr. Yu in writing and inform her that you withdraw your authorization to use your blood and intraocular fluid in future research.Her mailing address is Department of Ophthalmology, Beijing Hospital, 1 Dahua Road, Dongdan, Dongcheng District, Beijing.At that time, we will ask you to inform us in writing whether you require us to destroy your unused blood or allow your sample (which has been stripped of identifying information that can be traced to you) to be used in another study.

**17. Who should I contact if I have questions or difficulties?**

If you have any questions related to this study, please contact Dr. Yu at 010-85132171 or 15699847843 during office hours and 15699847843 after hours, weekends, and holidays.

If you have any questions related to your rights/interests, or if you would like to express your difficulties, grievances, or concerns about participating in this study, or if you would like to provide comments or suggestions related to this study, please contact the Ethics Committee of Beijing Hospital at 010-85138105 or bjyyec@126.com.

# Informed consent statement

I have been informed about the background, objectives, procedures, risks, and benefits of a randomized, controlled trial of micropulse laser plus ranibizumab in the treatment of diabetic macular edema.I have had enough time and opportunity to ask questions, and I am satisfied with the answers.I was also told who to contact if I had a question, a difficulty, a concern, a suggestion for the research, or if I wanted to obtain further information or help with the research.I have read the informed consent form and agree to participate in the study.I know that I could withdraw from the study at any time during the study without any reason.I was told that I would receive a copy of this signed and dated informed consent from myself and the investigator.

Subject Signature　　　　　　　　　　　　date

________　　　　　　　　　　　　________

Signature of Legal representative　　　　　　　　　　date

________　　　　　　　　　　　　________

# Investigator Notification Statement

I informed the subject of the background, objectives, procedures, risks, and benefits of a randomized controlled trial of micropulse laser combined with ranibizumab in the treatment of diabetic macular edema. I gave him/her enough time to read the informed consent form, discuss it with others, and answer his/her questions about the study.I have informed the subject that he can contact Dr. Yu at any time when he has any questions related to the study, and the Ethics Committee of Beijing Hospital at any time when he has any questions related to his own rights and interests, and provided the accurate contact information.The subject was informed that he/she could withdraw from the study;I informed the subject that he or she would receive a copy of this signed and dated informed consent form from the subject and me.

Signatures of researchers who obtained informed consent　　　　　　Date

________　　　　　　　　　　　　________
